# Immune Response Against Recent Omicron Sub-Lineages in Persons with HIV Receiving a Protein-Based or mRNA XBB.1.5 SARS-CoV-2 Booster Vaccine

**DOI:** 10.3390/ijms26083521

**Published:** 2025-04-09

**Authors:** Alessandra Vergori, Giulia Matusali, Eleonora Cimini, Alessandro Cozzi Lepri, Valentina Mazzotta, Davide Mariotti, Francesca Colavita, Simona Gili, Flavia Cristofanelli, Marisa Fusto, Roberta Gagliardini, Jessica Paulicelli, Federico Cecilia, Enrico Girardi, Fabrizio Maggi, Andrea Antinori

**Affiliations:** 1 Viral Immunodeficiency Unit, National Institute for Infectious Diseases L. Spallanzani, IRCCS, 00149 Rome, Italy; alessandra.vergori@inmi.it (A.V.); valentina.mazzotta@inmi.it (V.M.); marisa.fusto@inmi.it (M.F.); roberta.gagliardini@inmi.it (R.G.); jessica.paulicelli@inmi.it (J.P.); federico.cecilia@inmi.it (F.C.); andrea.antinori@inmi.it (A.A.); 2 Virology Unit, National Institute for Infectious Diseases L. Spallanzani, IRCCS, 00149 Rome, Italy; davide.mariotti@inmi.it (D.M.); francesca.colavita@inmi.it (F.C.); fabrizio.maggi@inmi.it (F.M.); 3 Laboratory of Cellular Immunology and Pharmacology, National Institute for Infectious Diseases L. Spallanzani, IRCCS, 00149 Rome, Italy; eleonora.cimini@inmi.it (E.C.); simona.gili@inmi.it (S.G.); flavia.cristofanelli@inmi.it (F.C.); 4 Institute of Global Health, University College London, CREME Center, London KT1 2EE, UK; a.cozzi-lepri@ucl.ac.uk; 5 Infectious Disease Unit, Department of Systems Medicine, University of Rome Tor Vergata, 00133 Rome, Italy; 6 Scientific Direction, National Institute for Infectious Diseases L. Spallanzani, IRCCS, 00149 Rome, Italy; enrico.girardi@inmi.it

**Keywords:** COVID-19, PWH, neutralizing response, SARS-CoV-2 XBB.1.5 vaccine, immunogenicity

## Abstract

The new Nuvaxovid protein-based and Pfizer-BioNTech mRNA-based vaccines targeting Omicron XBB.1.5 were available during the 2023–2024 autumn/winter vaccination campaign for frail individuals, including people with HIV (PWH). We assessed the immune response in 51 PWH on stable ART who received a booster with either the Nuvaxovid protein-based (*n* = 25) or Pfizer-BioNTech mRNA-based XBB.1.5 vaccine (*n* = 26). The median age was 57 years (IQR 51–65), the median count of CD4 at T0 was 652/mmc (503–935), and CD4 nadir was 226/mmc (95–340). Samples were collected before (T0) and one month after (T1) the booster. We measured neutralizing antibodies (nAbs) titers against D614G, XBB.1.6, and JN.1 variants and T-cell IFN-γ levels produced upon specific stimulation. Regardless of the vaccine used, we observed a marked increase in nAbs titers from T0 to T1 against all the subvariants, but no evidence for a change in IFN-γ release. After controlling for confounders, there was no evidence for a difference in the T0-T1 change in nAbs titers against XBB.1.16 and JN.1 by the type of vaccine, while Nuvaxovid determined a smaller increase in D614G nAbs (*p* = 0.008). The XBB.1.5 protein-based vaccine’s immunogenicity as a fifth or later booster was comparable to the Pfizer-BioNTech mRNA vaccine, particularly against recent Omicron variants.

## 1. Introduction

The COVID-19 pandemic has had a significant impact on global life expectancy, with variations across different waves and virus variants. In particular, the emergence of the Omicron variant influenced life expectancy trends compared to previous variants, such as limiting access to healthcare, and enhancing social or psychological stress. Omicron spread posed significant challenges to global public health due to its increased transmissibility and ability to partially evade immunity from previous infections and vaccinations.

A study found that the Omicron variant had higher transmission rates than Delta in England, with increased secondary attack rates in both household (15.0% vs. 10.8%) and non-household settings (8.2% vs. 3.7%). Vaccination reduced transmission, but the effect was weaker for Omicron. National data also showed a 3.5-fold higher risk of household clustering for Omicron, suggesting Omicron’s higher transmissibility and reduced vaccine effectiveness in reducing the risk of onward transmission contributing to its rapid spread [1].

Additionally, Omicron became dominant in Italy by December 2021–January 2022, representing 76.9–80.2% of cases. Its doubling time was 2.7–3.3 days, while Delta dropped to <6%. Despite Omicron’s faster spread, its lower severity is likely due to immunity from vaccination and previous infections [2].

Among the various sub-lineages of Omicron, XBB.1.5 has garnered particular attention due to its rapid spread and potential impact on vaccine efficacy.

Nuvaxovid XBB.1.5 is a new monovalent protein-based vaccine specifically designed to target the Omicron XBB.1.5 subvariant. This vaccine represents an important advancement in the ongoing efforts to enhance immune protection against evolving SARS-CoV-2 variants. Unlike traditional mRNA vaccines, protein-based vaccines like Nuvaxovid util1ize recombinant protein technology to elicit an immune response, which may offer distinct advantages in terms of stability and storage; e.g., mRNA vaccines (Pfizer-BioNTech and Moderna) require ultra-cold storage at −90 °C to −60 °C for long-term storage, and can be kept at 2 °C to 8 °C for up to 30 days after thawing [3]. In contrast, Nuvaxovid (Novavax) should be stored at 2 °C to 8 °C, and is stable for up to 9 months [4], and this means that a protein-based vaccine (Nuvaxovid) offers logistical advantages as it can be stored at standard refrigeration temperatures (2 °C–8 °C), simplifying distribution, especially in areas with limited resources instead of mRNA vaccines, which require a more complex cold chain with lower temperatures. Recent studies have demonstrated the reactogenicity and immunogenicity of monovalent XBB.1.5 mRNA vaccines, highlighting their ability to induce robust neutralizing antibody responses [5,6]. However, the efficacy of the Nuvaxovid XBB.1.5 vaccine in boosting immune responses has primarily been inferred from data on the original Wuhan strain and the adapted Omicron BA.5-strain vaccine [7,8,9]. To date, only one study has investigated the effectiveness of monovalent XBB.1.5 vaccines against more recent circulating variants, in which the protein-based Nuvaxovid is included. Indeed, the effectiveness in reducing the risk of SARS-CoV-2 infection was globally higher after the first 4 weeks from the booster, and then gradually decreased from 6 weeks post-booster, regardless of age, sex, race and ethnicity, socioeconomic status, or previous immunity status.

Overall, XBB.1.5 mRNA vaccines were effective against omicron subvariants, although less so against JN.1, although the effectiveness of the Novavax vaccine could not be reliability estimated due to the limited number of recipients [10]. Furthermore, specific data on the immunogenicity of the protein-based XBB.1.5 vaccine in people living with HIV (PWH) are lacking, despite the known vulnerabilities of this population to COVID-19 [11].

Evidence suggests that PWH develop immune responses to COVID-19 vaccines, but these vary based on CD4 count, viral load, and vaccine type [12]. PWH with well-controlled HIV show similar immunogenicity to HIV-negative individuals, while those with CD4 < 200 cells/µL or unsuppressed viremia have weaker responses [13]. Boosters enhance immunity, especially in those with suboptimal initial responses, and mRNA vaccines are more effective than viral vector vaccines [14].

On 4 December 2023, the Italian Ministry of Health recommended the use of Nuvaxovid XBB.1.5 as an alternative to the Pfizer-BioNTech mRNA vaccine XBB.1.5 for the 2023–2024 autumn/winter COVID-19 vaccination campaign, particularly for frail individuals starting from the age of 12, including PWH [15]. This recommendation underscores the need for comprehensive studies to evaluate the comparative immunogenicity and efficacy of these vaccines in vulnerable populations.

This study aims to fill this gap by assessing the immune response in PWH who received either the Nuvaxovid protein-based or Pfizer-BioNTech mRNA XBB.1.5 booster vaccine. By measuring neutralizing antibody titers and T-cell responses, we seek to provide insights into the ability of these vaccines to enhance immunity against XBB.1.16 and JN.1 Omicron sub-lineages in this high-risk group.

## 2. Results

We included 51 PWH (*n* = 26 received the Pfizer-BioNTech mRNA and *n* = 25 the Nuvaxovid XBB.1.5). The median age was 57 years (Interquartile range, IQR 51, 65). The 65%, 25%, and 10% had previously received 5, 4, and 6 vaccine doses, respectively (Table 1).

**Figure 1:**
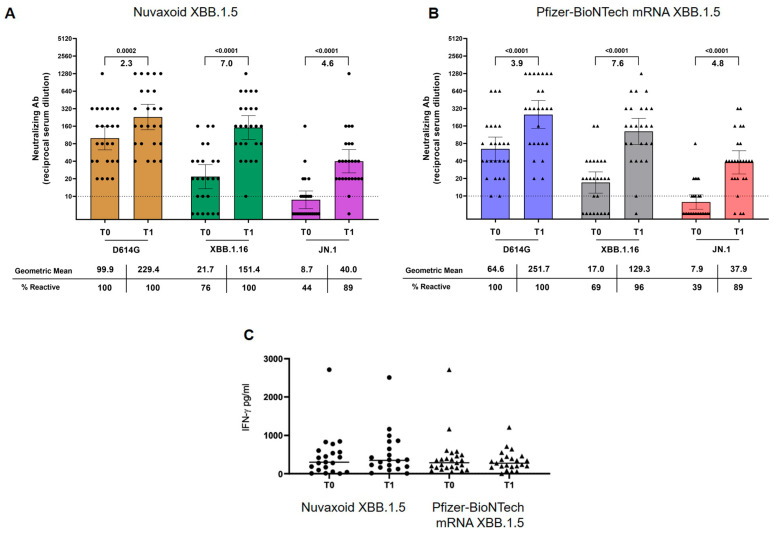
Neutralizing and T cell response levels in PWH receiving XBB.1.5 vaccines. Geometric means neutralizing antibodies titers (**A**,**B**) and median values of IFN-γ (**C**) at T0 and T1, stratified by intervention. (**A**,**B**) *p*-values of Wilcoxon test and the fold change obtained comparing nAbs at T0 and T1 are indicated above and below the horizontal square bracket, respectively. Dashed lines represent the limit of the detection of the assay, error bars indicate the 95% confidence interval.

Overall, 24/51 (47%) were positive for anti-N IgG before the vaccine booster, suggesting previous exposure to SARS-CoV-2 (natural infection), of whom 13/24 (54%) received Nuvaxovid XBB.1.5 and 11/24 (45%) the Pfizer-BioNTech mRNA; among those who tested negative for anti-N IgG at T0, 2 tested positive at T1 (1 received Nuvaxovid XBB.1.5 and 1 Pfizer-BioNTech mRNA). Regardless of the vaccine used, over T0–T1, we observed a marked increase in anti-RBD IgG (*p* < 0.0001) and nAbs titers against all the subvariants (D614G *p* < 0.0001; XBB.1.16 *p* < 0.0001; JN.1 *p* < 0.0001), but no evidence for a change in IFN-γ release (Figure 1).

After controlling for confounders in the double robust analysis, we found evidence that PWH receiving the Nuvaxovid XBB.1.5 vaccine showed smaller mean log_2_ changes over T0–T1 of anti-RBD IgG than participants boosted with the Pfizer-BioNTech mRNA. Using the double robust method potential outcome were 1.12 log_2_ (95% CI: 0.20, 2.05) vs. 2.15 log_2_ (95% CI: 1.05, 3.26), with a corresponding average treatment effect (ATE) of −1.03 (−1.79, −0.27); *p* = 0. 008]. Similarly, changes in nAbs titers against D614G were smaller for Nuvaxovid than for Pfizer-BioNTech mRNA [ATE −1.31 (−2.16, −0.47) *p* = 0.002]. There was no evidence for a difference in nAbs titers by vaccination type against the other variants investigated (Table 2).

## 3. Discussion

To our knowledge, this is the first study using causal inference analysis to compare the in vivo immunogenicity of monovalent XBB.1.5 protein-based vs. Pfizer-BioNTech mRNA vaccines in PWH. Our data carried little evidence for a difference in the ability of the Nuvaxovid XBB.1.5 and Pfizer-BioNTech XBB.1.5 mRNA vaccines to boost neutralizing responses against the newly circulating SARS-CoV-2 variants, XBB.1.5 and JN.1. Both vaccine formulations induced a marked increase in anti-RBD IgG and geometric mean titers (GMT) of neutralizing antibodies against the XBB.1.5 variant, as well as the more recent JN.1 variant. It has been previously established that the XBB.1.5-adapted mRNA vaccine significantly boosts plasma neutralization potency against the XBB.1.5 variant, suggesting its likely effectiveness in protecting against severe infections from currently circulating SARS-CoV-2 strains [16]. This aligns with recent epidemiological studies demonstrating the effectiveness of the XBB.1.5-adapted vaccine against various Omicron subvariants. The increase in neutralizing titers observed with both vaccines appears to be primarily driven by the reactivation of pre-existing B cell memory [10,17]. Interestingly, despite the overall similarity in the immune responses induced by both vaccines against the XBB.1.5 and JN.1 strains, the Pfizer-BioNTech XBB.1.5 mRNA vaccine induced higher levels of anti-RBD IgG and nAbs against the D614G strain compared to Nuvaxovid. This difference appears to be consistent with the findings from a recent randomized, non-inferiority trial (Com-COV2), which showed that the second dose of an mRNA vaccine was able to stimulate a greater humoral response than the protein-based vaccine NVX-CoV2373, although responses to the second vs. fourth dose are not strictly comparable [18]. In contrast, our analysis did not support the superiority of the Pfizer-BioNTech vaccine over Nuvaxovid when measured against the JN.1 strain, i.e., the Omicron sub-lineage responsible for most infections detected in 2024 [19]. Concerning the T-cell response, the current analysis confirms our previous findings [20]. It suggests that newly developed Omicron-targeted vaccines (like their predecessors) are unlikely to further increase the T-cell immunity achieved with the primary cycle plus a single booster of vaccinations or with natural infection. A limitation of the study is the small sample size and the short follow up period which prevented us from drawing firm conclusions on the persistence or waning of vaccine induced immune response. Of note, another limitation is the non-availability of neutralization data against the increasingly circulating KP.2, KP.3, and XEC subvariants. However, compared to JN.1, these more recent sub-lineages have demonstrated a similar reduced susceptibility to neutralization by sera from individuals vaccinated with anti-XBB.1.5 vaccines [21]. Most importantly, our analysis of the comparison of response by intervention is only valid under the usual assumptions of consistency, positivity, and exchangeability.

## 4. Materials and Methods

The HIV-VAC study is an observational, ongoing monocentric project on the surveillance of anti-SARS-CoV-2 vaccination in PWH with a wide range of CD4 count at time of receiving the booster vaccination, mainly in the CD4 > 350 cells/mm^3^ range [22]. A normal CD4 count ranges from 500 to 1500 cells/mm³, with higher values reflecting better immune function. A low CD4 count (<200 cells/mm³) weakens immunity, increasing the risk of infections and the risk of a poor vaccine response [13]. In contrast, higher CD4 counts provide better protection against infections and severe illness, and an immunological response to vaccines like that of the general population [13]. Details have been described elsewhere [16]. The study on PWH (HIV-VAC) was approved by the Scientific Committee of the Italian Drug Agency (AIFA) and by the Ethical Committee of the Lazzaro Spallanzani Institute (approval number: 423/2021; amendment adopted with no. 91/2022). The participants included in the present study provided informed written consent. Samples were collected before (day 0, T0) and one month after the booster dose with Nuvaxovid or the Pfizer vaccines (T1). The type of vaccine was pseudo-randomly allocated based on the availability of the doses on the day of the visit. Anti-receptor binding domain (RBD) IgG (binding antibody units (BAU)/mL, positive if ≥7.1) and anti-nucleocapsid (N) IgG (sample (S)/Cutoff (CO), positive if ≥1.4) were tested by a chemiluminescent microparticle immunoassay (CMIA) on the Abbott Alinity i platform. Neutralizing antibody titers against the SARS-CoV-2 strain D614G, XBB.1.16 (XBB.1.5 like variant), and JN.1 were measured by a micro-neutralization assay based on a live virus, as previously described [23]. Briefly, serum samples were inactivated at 56 °C for 30 min, diluted 1:10 in serum-free minimum essential medium, and two-fold serially diluted to 1:640. One hundred TCID50 SARS-CoV-2 were mixed with serum dilutions and incubated at 37 °C, 5% CO_2_ for 30 min. Subsequently, serum–virus mixtures were incubated on Vero E6 cell monolayers at 37 °C and 5% CO_2_. The endpoint titer of neutralizing antibodies was established by highly experienced personnel to assess the cytopathic effect. The highest serum dilution inhibiting at least 90% of the cytopathic effect was defined as neutralizing. The sequences of the SARS-CoV-2 isolate used in microneutralization assays are available on GISAID: D614G hCoV-19/Italy/LOM-INMI-10734/2020 EPI_ISL_568579; XBB.1.16 strain SARS-CoV-2/human/ITA/LAZ-INMI-5327/2023 EPI_ISL_18746183; JN.1 strain hCoV-19/Italy/LAZ-INMI-5946/2023 EPI_ISL_18673911. The IFN-γ release from T-cell-specific response was analyzed by an ELISA test after Spike–peptide stimulation [24]. The detection limit of this assay was 0.17 pg/mL and the cut off used to define the T-cells specific responses was 12 pg/mL. The geometric means of the nAbs titers (GMTs) and median (IQR) IFN-γ values over T0–T1 were calculated and levels compared by the Wilcoxon test. Comparison tests were performed using GraphPad Prism version 10.0.0 for Windows (GraphPad Software, Boston, MA, USA).

The average treatment effect (ATE) was estimated by regression adjustment and predicted values for the potential outcomes (counterfactual linear marginal model), and for the comparison of the changeover T0–T1 in anti-RBD IgG levels, nAbs titers, and IFN-γ values (expressed in a log2 scale) under the intervention (Nuvaxovid XBB.1.5) or control (Pfizer-BioNTech mRNA XBB.1.5) after weighting for potential confounders, including age, current and nadir CD4 count, year of last booster before XBB monovalent vaccine, year of HIV diagnosis, days from previous vaccine dose, number of doses previously received and positivity of anti-N IgG at T0. We used inverse probability weighting (IPW), regression adjustment and a double robust approach with the same linear predictors for both the propensity and outcome model (augmented IPW) to calculate average treatment effects (ATE). Doubly robust estimation methods fit models for both the outcome and the treatment variables. They combine inverse probability weighting and regression adjustment to estimate the potential outcome means. The methods are said to be doubly robust because they provide unbiased estimates even if one of the two models is mis-specified [25]. In this sense, you have two opportunities to obtain an unbiased estimation of the causal effect by specifying either a correct treatment or a correct outcome model. For this analysis, doubly robust estimation was implemented using the augmented inverse probability weighting (AIPW) method, as previously described [26]. With “counterfactual linear marginal model”, we mean that the average treatment effect (ATE) was estimated by regression adjustment, and predicted values for the potential outcomes were calculated. In more detail, we predicted the potential outcomes from generalized linear models that were fit to the data using maximum likelihood estimation. A potential outcome is the outcome for a participant under a potential treatment. For this participant, the causal effect of the treatment is the difference between the potential outcome if the individual had received the protein-based and the potential outcome if the individual had received the mRNA XBB.1.5 SARS-CoV-2 booster vaccine instead (only one of these is observed, the other is predicted). Because one of the potential outcomes is missing data, the ATE is calculated at population level. SAS v9.4 (Cary, NC, USA) was used for the main statistical analysis (SAS Institute Inc., SAS 9.1.3 Help and Documentation, Cary, NC, USA: SAS Institute Inc., 2002–2004).

## 5. Conclusions

In conclusion, our analysis suggests that in our sample of PWH, the immunogenicity elicited by the XBB.1.5 protein-based vaccine when used as a fifth or later booster was comparable to that achieved using a Pfizer-BioNTech mRNA vaccine at 1 month post-vaccination against the Omicron XBB.1.6 and JN.1 subvariants of SARS-CoV-2. These data might help guide public health policy and plan vaccination strategies for PWH.

## Figures and Tables

**Table 1:** Descriptive characteristics of study population according to type of vaccine received.

	Type of Vaccine			
Characteristic	Nuvaxovid XBB.1.5 *n* = 25	Pfizer-BioNTech mRNA XBB.1.5 *n* = 26	*p*-Value *	Total *N* = 51
Age, years, median (IQR)	60 (52, 64)	55 (51, 65)	0.497	57 (51, 65)
CD4 count at T0, cells/mm^3^, median (IQR)	622 (503, 771)	783 (515, 976)	0.296	652 (503, 935)
Nadir CD4 count, cells/mm^3^, median (IQR)	212 (26, 298)	297 (148, 362)	0.083	226 (95, 340)
Year of HIV diagnosis, median (IQR)	2007 (2004, 2011)	2008 (2004, 2011)	0.765	2008 (2004, 2011)
Year of current booster dose (post 4th dose), median (IQR)	2022 (2021, 2022)	2022 (2022, 2022)	0.637	2022 (2022, 2022)
Days between boost and T0, median (IQR)	450 (423, 766)	447 (378, 631)	0.243	450 (402, 656)
Days between T0 and T1, median (IQR)	32 (29, 36)	30 (28, 33)	0.127	30 (28, 35)
Previous vaccination (no. doses), n (%)			0.450	
4	8 (32.0)	5 (19.2)		13 (25.5)
5	14 (56.0)	19 (73.1)		33 (64.7)
6	3 (12.0)	2 (7.7)		5 (9.8)
Humoral immunity at T0, median (IQR)				
anti-N IgG (S/CO)	1.3 (0.2, 4.0)	0.5 (0.1, 7.1)	0.955	0.8 (0.1, 5.8)
anti-RBD IgG (BAU/mL)	1661 (779.6, 3361)	1861 (859.7, 3266)	0.992	1727 (779.6, 3361)
nAbs against D614G	80.0 (40.0, 320.0)	60.0 (40.0, 160.0)	0.171	80.0 (40.0, 160.0)
nAbs against XBB.1.6	20.0 (10.0, 40.0)	20.0 (5.0, 40.0)	0.462	20.0 (5.0, 40.0)
nAbs against JN.1	5.0 (5.0, 10.0)	5.0 (5.0, 10.0)	0.702	5.0 (5.0, 10.0)
Humoral immunity at T1, median (IQR)				
anti-N IgG (S/CO)	1.5 (0.2, 2.2)	0.5 (0.1, 5.6)	0.962	1.2 (0.2, 5.5)
anti-RBD IgG (BAU/mL)	4232 (2008, 8462)	6942 (4804, 11,360)	0.027	5160 (3481, 11,360)
nAbs against D614G	160.0 (80.0, 640.0)	320.0 (80.0, 1280)	0.716	320.0 (80.0, 640.0)
nAbs against XBB.1.16	160.0 (80.0, 320.0)	160.0 (80.0, 320.0)	0.752	160.0 (80.0, 320.0)
nAbs against JN.1	40.0 (20.0, 80.0)	40.0 (20.0, 80.0)	0.846	40.0 (20.0, 80.0)
T-cell immunity, median (IQR)				
IFN-γ at T0 (pg/mL)	301.4 (96.6, 564.7)	286.8 (156.8, 461.1)	0.886	294.1 (150.5, 535.0)
IFN-γ at T1 (pg/mL)	347.6 (175.9, 746.4)	272.7 (180.8, 445.7)	0.394	315.3 (180.8, 485.7)

* Chi-square or Mann–Whitney test as appropriate. Abbreviations: anti N, anti-nucleocapside; S/CO, sample/Cutoff; anti-RBD, anti-receptor binding domain; BAU/mL, binding antibody units per milliliter; nAbs, neutralizing antibodies expressed as a reciprocal of serum dilution.

**Table 2:** Potential average change over T0–T1 and ATE from fitting a linear regression model (log_2_ scale).

	Potential Average Change at Post-Vaccine Dose and ATE ^&^ from Fitting a Linear Regression Model (log_2_ Scale)			
	Mean (log_2_) in Nuvaxovid XBB.1.5 (95% CI)	Mean (log_2_) in Pfizer mRNA XBB.1.5 (95% CI)	ATE * (95% CI)	*p*-Value
*Causal inference method*	*Anti-RBD*			
IPWs	1.15 (0.77, 1.53)	2.25 (1.56, 2.95)	−1.10 (−1.88, −0.33)	0.005
Double robust (AIPW)	1.12 (0.20, 2.05)	2.15 (1.05, 3.26)	−1.03 (−1.79, −0.27)	0.008
Regression adjustment	1.15 (0.69, 1.61)	2.11 (1.11, 3.10)	−0.96 (−2.03, 0.12)	0.080
	*nAbs against D614G*			
IPWs	1.19 (0.82, 1.56)	2.70 (1.82, 3.57)	−1.51 (−2.44, −0.58)	0.001
Double robust (AIPW)	1.15 (0.50, 1.81)	2.46 (0.90, 4.03)	−1.31 (−2.16, −0.47)	0.002
Regression adjustment	1.18 (0.72, 1.65)	2.41 (1.28, 3.53)	−1.22 (−2.42, −0.02)	0.046
	*nAbs against XBB.1.16*			
IPWs	2.99 (2.39, 3.60)	3.39 (2.47, 4.31)	−0.39 (−1.45, 0.67)	0.466
Double robust (AIPW)	3.05 (1.85, 4.26)	3.12 (1.56, 4.68)	−0.06 (−1.03, 0.91)	0.899
	*nAbs against JN.1*			
IPWs	2.21 (1.78, 2.63)	2.52 (2.03, 3.01)	−0.31 (−0.94, 0.32)	0.332
Double robust (AIPW)	2.13 (1.51, 2.75)	2.36 (1.30, 3.41)	−0.23 (−0.84, 0.39)	0.473
	*IFN-γ*			
IPWs	0.59 (0.13, 1.04)	0.11 (−0.62, 0.83)	0.48 (−0.39, 1.35)	0.278
Double robust (AIPW)	0.61 (−0.28, 1.50)	0.25 (−3.70, 4.19)	0.37 (−0.85, 1.58)	0.556
Regression adjustment	0.62 (0.00, 1.24)	0.29 (−0.56, 1.14)	0.33 (−0.76, 1.42)	0.551

^&^ ATE, average treatment effect; IPW, inverse probability weighting; AIPW, augmented inverse probability weighting. * Weighted for age, current and nadir CD4 count, year of booster, year of HIV diagnosis, days from previous vaccine dose number of doses previously received and previous evidence of natural infection.

## Data Availability

The dataset will be available on reasonable request to the corresponding author.
